# Supplementary material for: Conformational Changes in Ff Phage Protein gVp upon Complexation with Its Viral Single-Stranded DNA Revealed Using Magic-Angle Spinning Solid-State NMR
Source: Viruses. 2022 Jun 10;14(6):1264. doi: 10.3390/v14061264 (PMC9231167; doi:10.3390/v14061264)
Supplement: Supplementary file 1 [file viruses-14-01264-s001.zip › viruses-1750529-supplementary/viruses-1750529-supplementary.pdf]

Supporting Information

Conformational changes in Ff phage protein gVp upon complexation with its viral single-stranded DNA revealed by magic-angle-spinning solid-state NMR

Smadar Kedem, Roni Rene Hassid, Yoav Shamir, Amir Goldbourt*

School of chemistry, Tel Aviv University, Ramat Aviv 6997801, Tel Aviv

Figure S1. Purification of gVp: Coomassie Blue on 18% SDS-PAGE gel. (1) Protein Ladder (10-250kD), (2) cells before induction of gVp, (3) cells after induction of gVp in minimal media, (4) purified gVp, after his-tag column purification and dialysis. The total protein yield averaged ~45.0 mg for a fully labeled sample and ~10.0 mg for a sparsely labeled sample, per 1 liter of the starting culture.

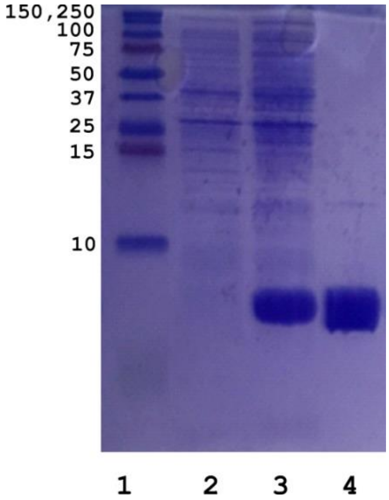

Figure S2. UV absorption spectra showing titration of fd-ssDNA into gVp at different complexation ratios (NPM: nucleotides per protein).

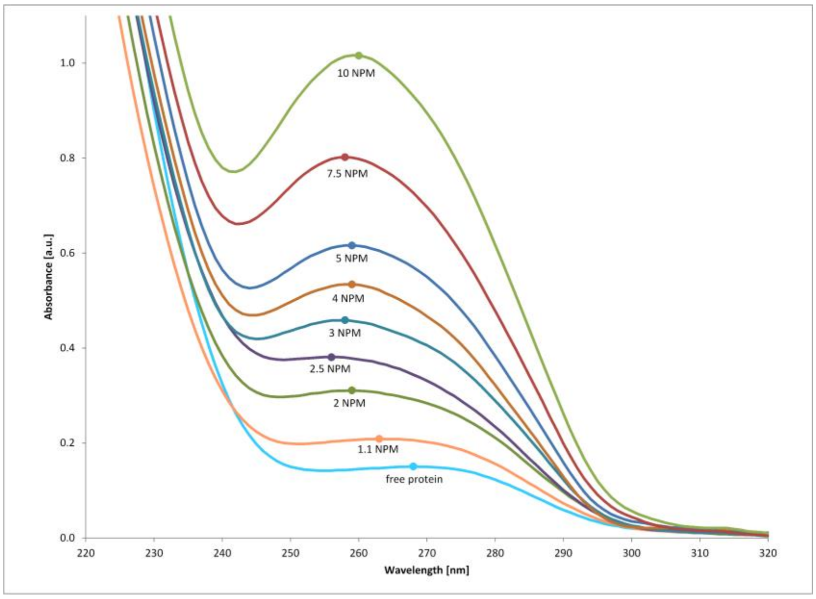

Figure S3. DARR100 of gly13-gVp-NA-DNA (blue) and U-gVp-NA-DNA (orange). The inset shows the correlation between T15 and R16 in the bound form (see the discussion of figure 8 regarding the large chemical shift difference of T15Cβ, bound vs free).

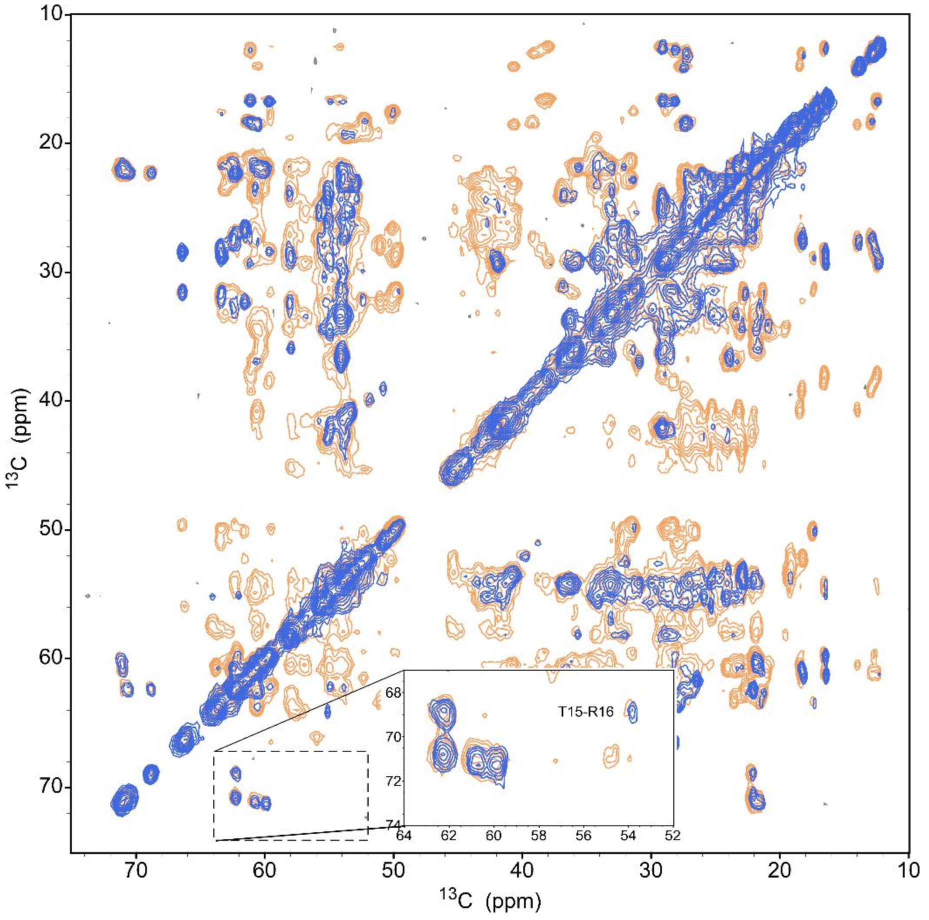

Figure S4. Assignment strip plots for the entire gVp protein. NCACX in blue, NCOCX in red. Wherever strips from the 3D experiments are not available, matching strips from 2D experiments are included.

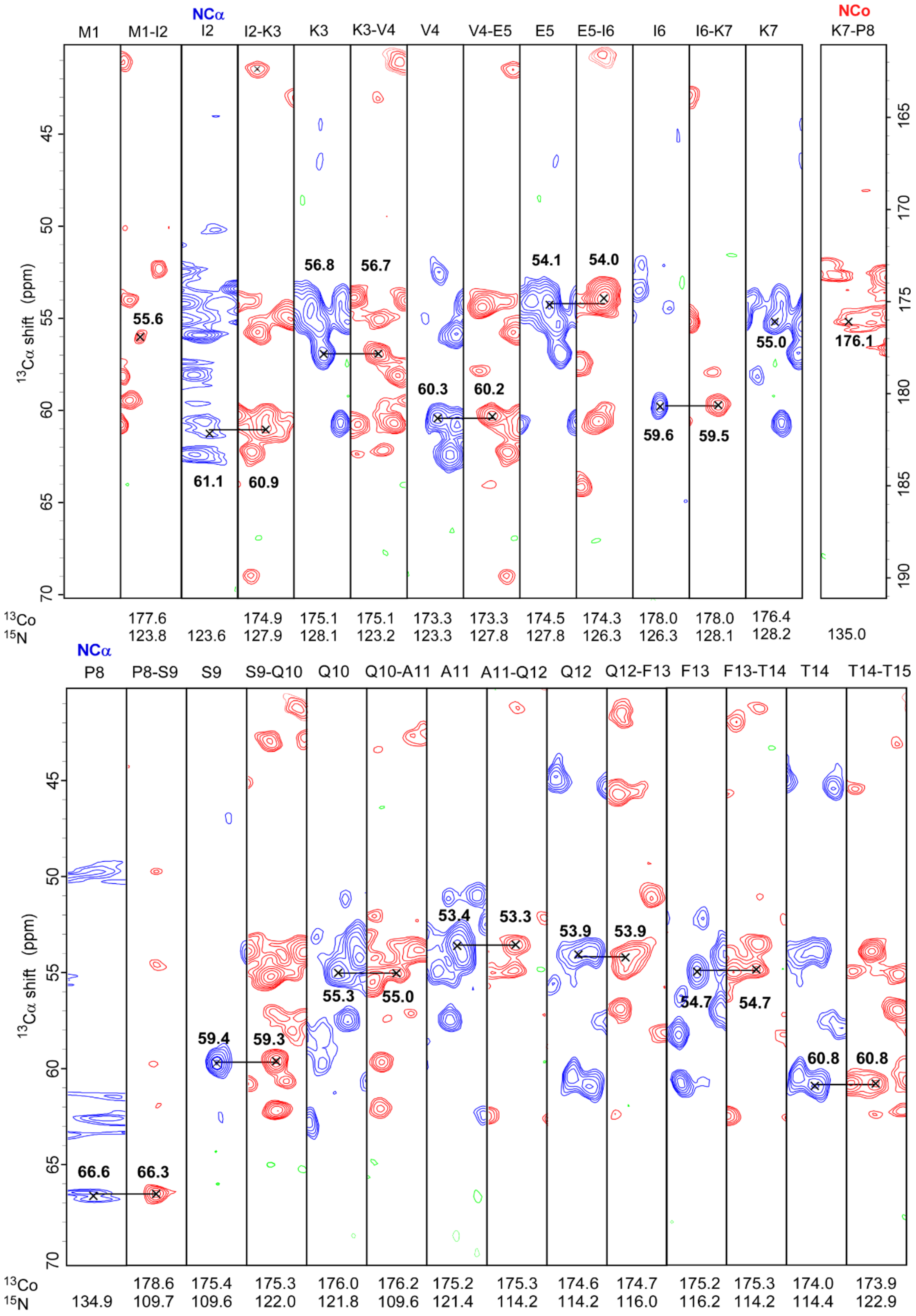

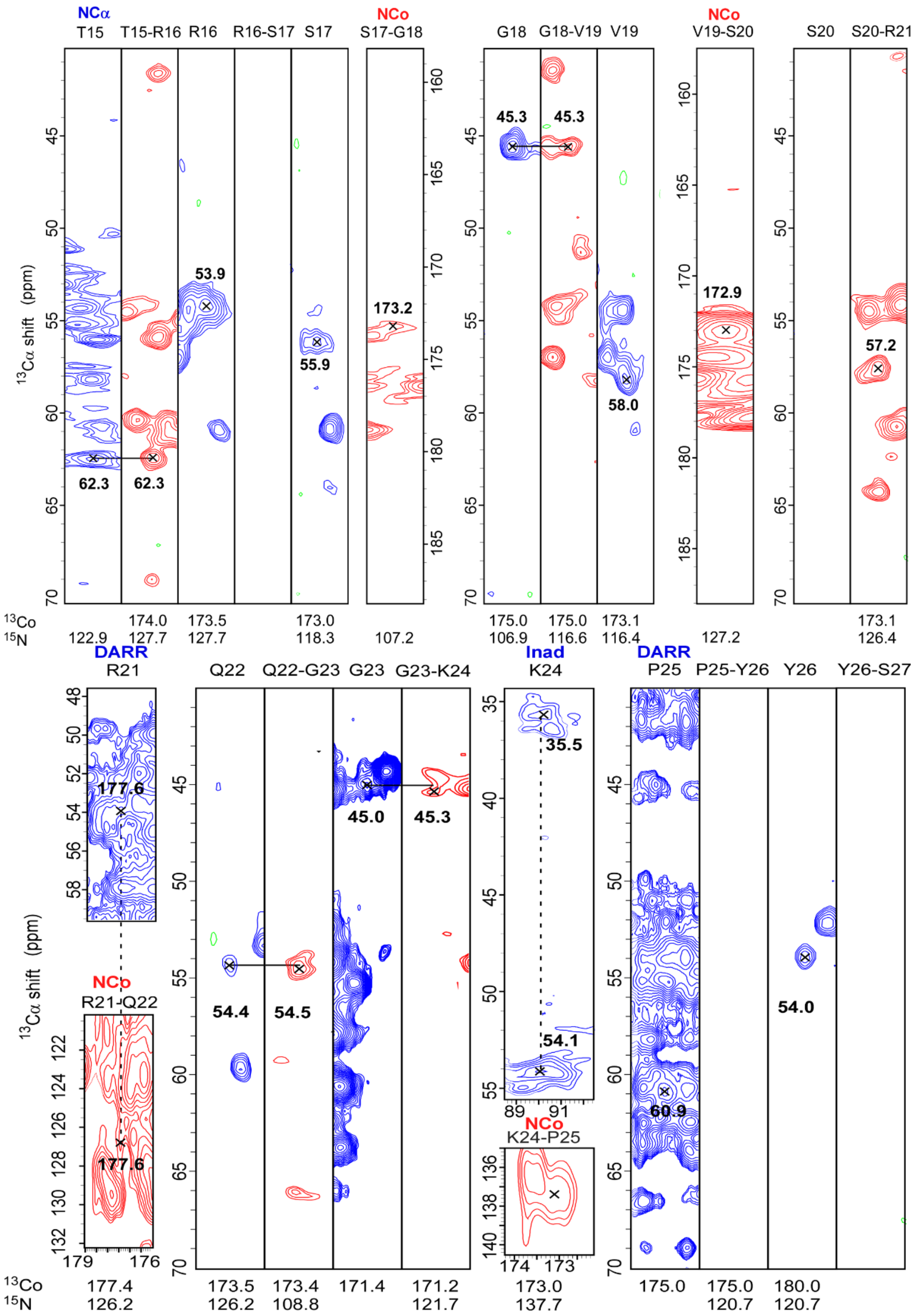

NCα
T15
T15-R16
R16
R16-S17
S17
NCo
S17-G18
G18
G18-V19
V19
NCo
V19-S20
S20
S20-R21
13Cα shift (ppm)
53.9
55.9
62.3
62.3
173.2
45.3
45.3
58.0
172.9
57.2
13Co
15N
122.9
174.0
127.7
173.5
127.7
173.0
118.3
107.2
175.0
106.9
175.0
116.6
173.1
116.4
127.2
173.1
126.4
DARR
R21
Q22
Q22-G23
G23
G23-K24
Inad
K24
DARR
P25
P25-Y26
Y26
Y26-S27
177.6
NCo
R21-Q22
177.6
54.4
54.5
45.0
45.3
35.5
54.1
NCo
K24-P25
60.9
54.0
177.4
126.2
173.5
126.2
173.4
108.8
171.4
171.2
121.7
173.0
137.7
175.0
175.0
120.7
180.0
120.7

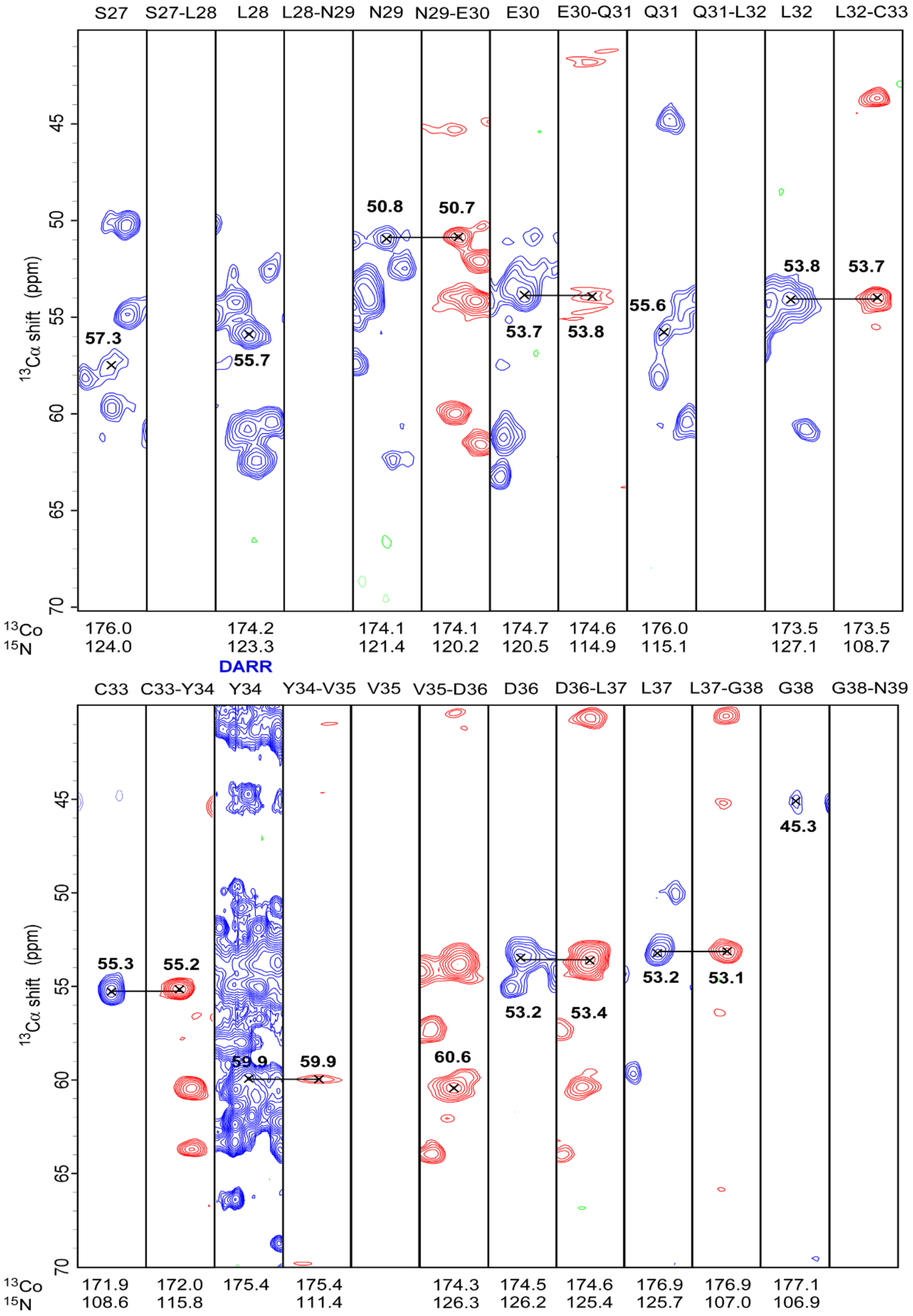
S27
S27-L28
L28
L28-N29
N29
N29-E30
E30
E30-Q31
Q31
Q31-L32
L32
L32-C33
57.3
55.7
50.8
50.7
53.7
53.8
55.6
53.8
53.7
$^{13}C\alpha$ shift (ppm)
45
50
55
60
65
70
^{13}Co
^{15}N
176.0
124.0
174.2
123.3
174.1
121.4
174.1
120.2
174.7
120.5
174.6
114.9
176.0
115.1
173.5
127.1
173.5
108.7
DARR
C33
C33-Y34
Y34
Y34-V35
V35
V35-D36
D36
D36-L37
L37
L37-G38
G38
G38-N39
55.3
55.2
59.9
59.9
60.6
53.2
53.4
53.2
53.1
45.3
171.9
108.6
172.0
115.8
175.4
175.4
111.4
174.3
126.3
174.5
126.2
174.6
125.4
176.9
125.7
176.9
107.0
177.1
106.9

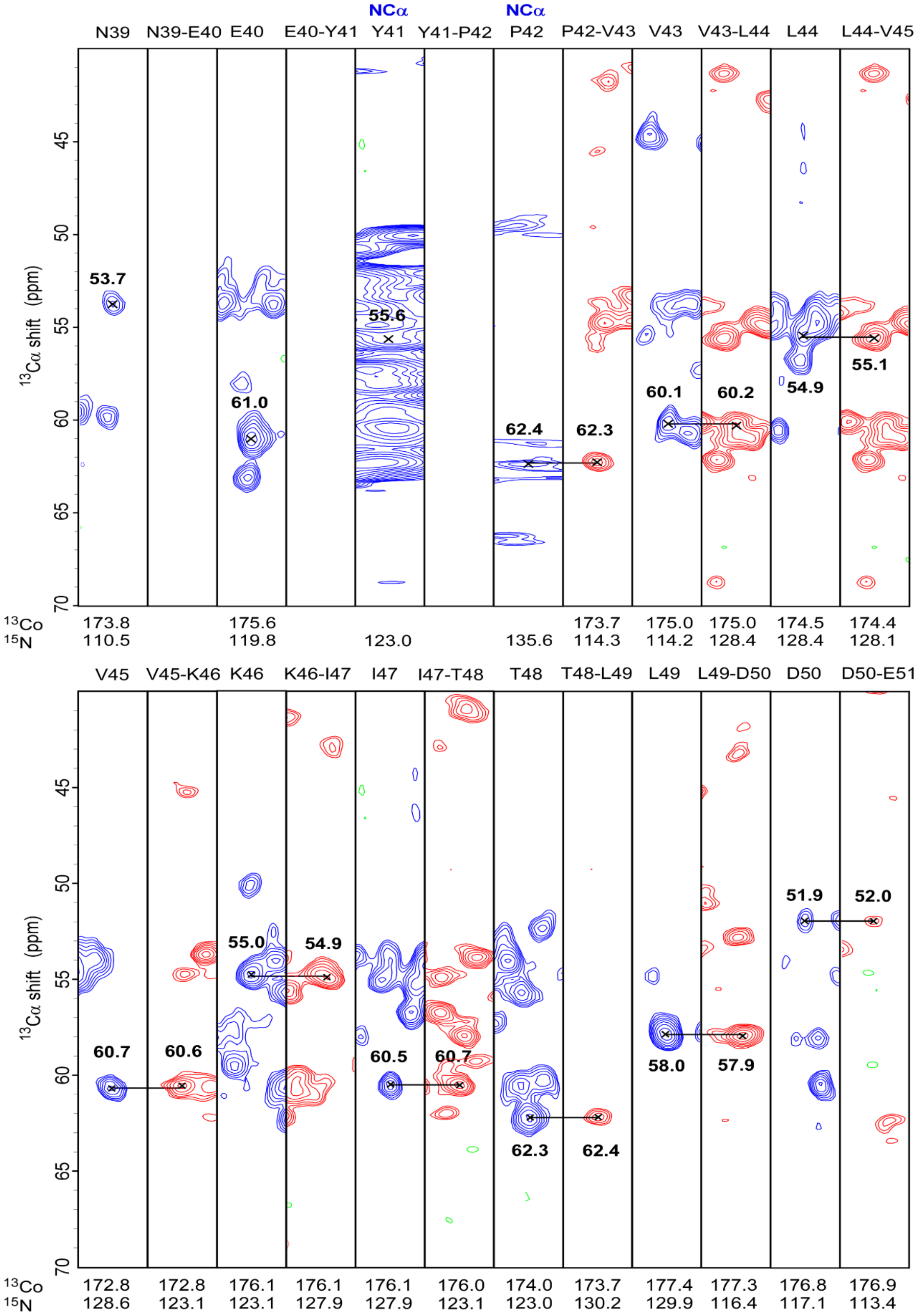

NCα
NCα
N39
N39-E40
E40
E40-Y41
Y41
Y41-P42
P42
P42-V43
V43
V43-L44
L44
L44-V45
13Cα shift (ppm)
53.7
61.0
55.6
62.4
62.3
60.1
60.2
54.9
55.1
13Co 173.8 175.6 173.7 175.0 175.0 174.5 174.4
15N 110.5 119.8 123.0 135.6 114.3 114.2 128.4 128.4 128.1
V45
V45-K46
K46
K46-I47
I47
I47-T48
T48
T48-L49
L49
L49-D50
D50
D50-E51
55.0
54.9
60.7
60.6
60.5
60.7
62.3
62.4
58.0
57.9
51.9
52.0
13Co 172.8 172.8 176.1 176.1 176.1 176.0 174.0 173.7 177.4 177.3 176.8 176.9
15N 128.6 123.1 123.1 127.9 127.9 123.1 123.0 130.2 129.9 116.4 117.1 113.4

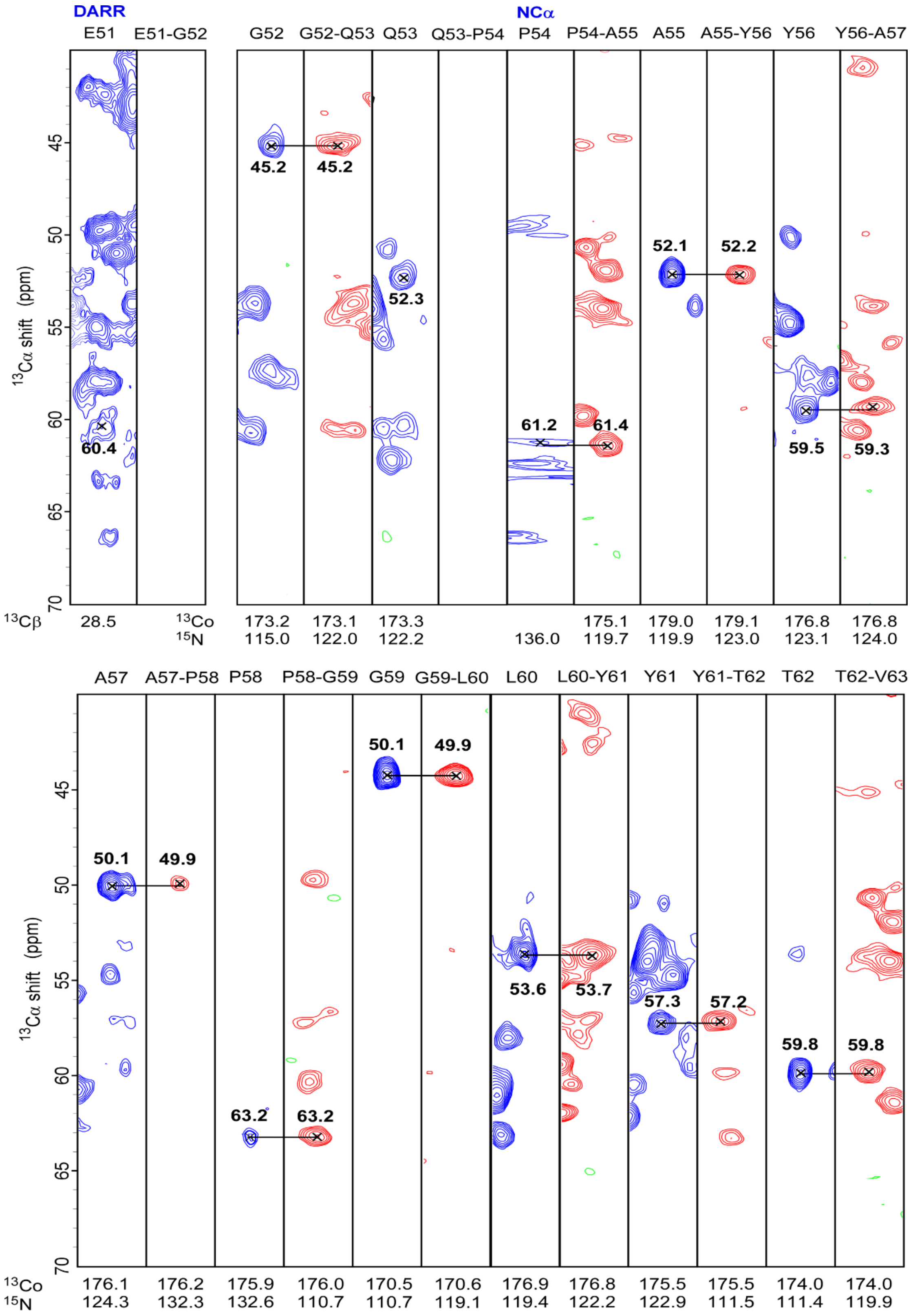

DARR
NCα
E51 E51-G52 G52 G52-Q53 Q53 Q53-P54 P54 P54-A55 A55 A55-Y56 Y56 Y56-A57
45.2 45.2 52.3 60.4 61.2 61.4 52.1 52.2 59.5 59.3
^{13}Cα shift (ppm)
^{13}Cβ 28.5
^{13}Co ^{15}N
173.2 115.0 173.1 122.0 173.3 122.2 136.0 175.1 119.7 179.0 119.9 179.1 123.0 176.8 123.1 176.8 124.0
A57 A57-P58 P58 P58-G59 G59 G59-L60 L60 L60-Y61 Y61 Y61-T62 T62 T62-V63
50.1 49.9 50.1 49.9 63.2 63.2 53.6 53.7 57.3 57.2 59.8 59.8
176.1 124.3 176.2 132.3 175.9 132.6 176.0 110.7 170.5 110.7 170.6 119.1 176.9 119.4 176.8 122.2 175.5 122.9 175.5 111.5 174.0 111.4 174.0 119.9

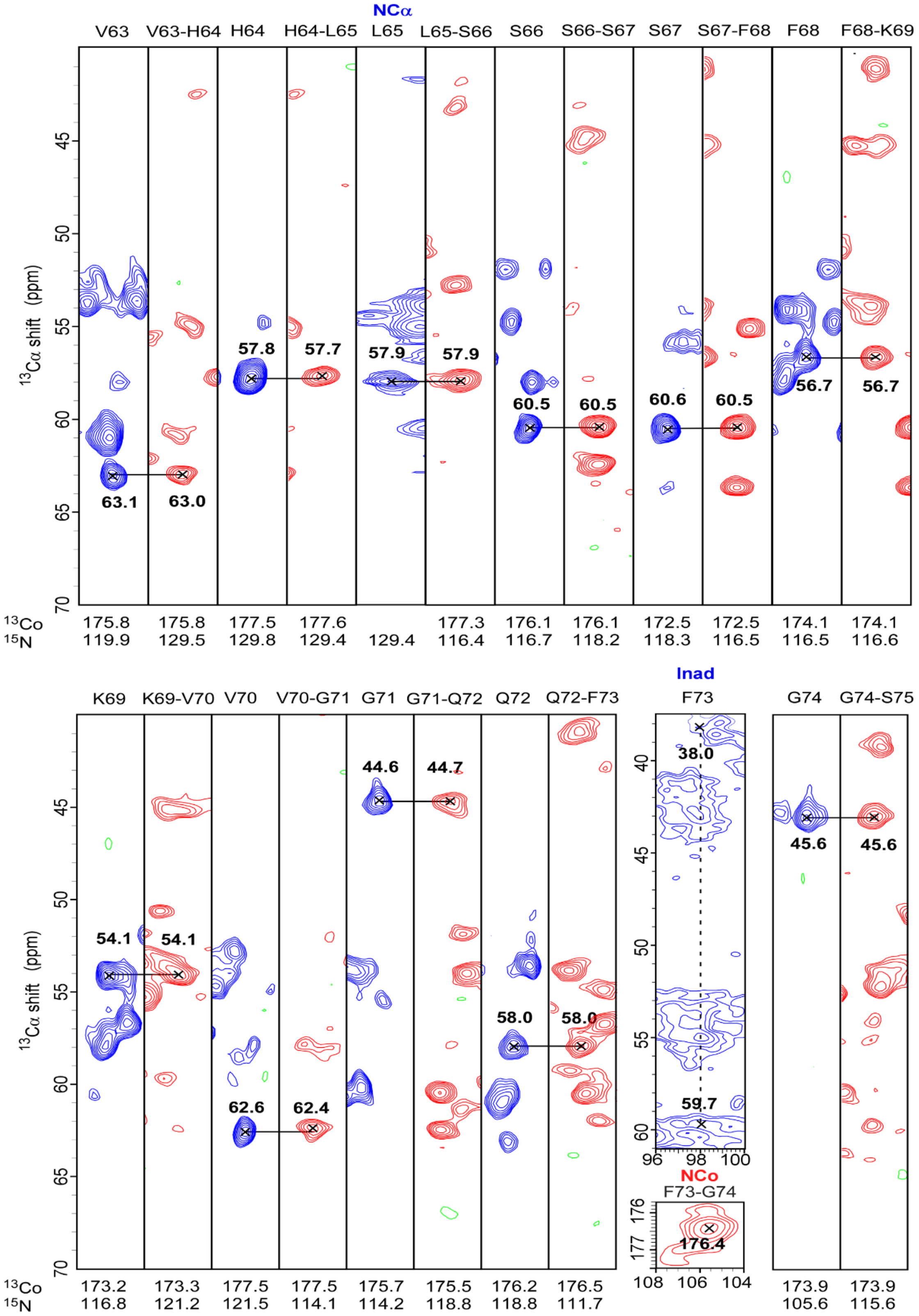

NCα
V63
V63-H64
H64
H64-L65
L65
L65-S66
S66
S66-S67
S67
S67-F68
F68
F68-K69
63.1
63.0
57.8
57.7
57.9
57.9
60.5
60.5
60.6
60.5
56.7
56.7
45
50
55
60
65
70
$^{13}C\alpha$ shift (ppm)
^{13}Co
^{15}N
175.8 119.9
175.8 129.5
177.5 129.8
177.6 129.4
129.4
177.3 116.4
176.1 116.7
176.1 118.2
172.5 118.3
172.5 116.5
174.1 116.5
174.1 116.6
Inad
K69
K69-V70
V70
V70-G71
G71
G71-Q72
Q72
Q72-F73
F73
G74
G74-S75
54.1
54.1
62.6
62.4
44.6
44.7
58.0
58.0
38.0
59.7
45.6
45.6
40
45
50
55
60
96
98
100
NCo
F73-G74
176
177
176.4
108
106
104
173.2 116.8
173.3 121.2
177.5 121.5
177.5 114.1
175.7 114.2
175.5 118.8
176.2 118.8
176.5 111.7
173.9 105.6
173.9 115.6

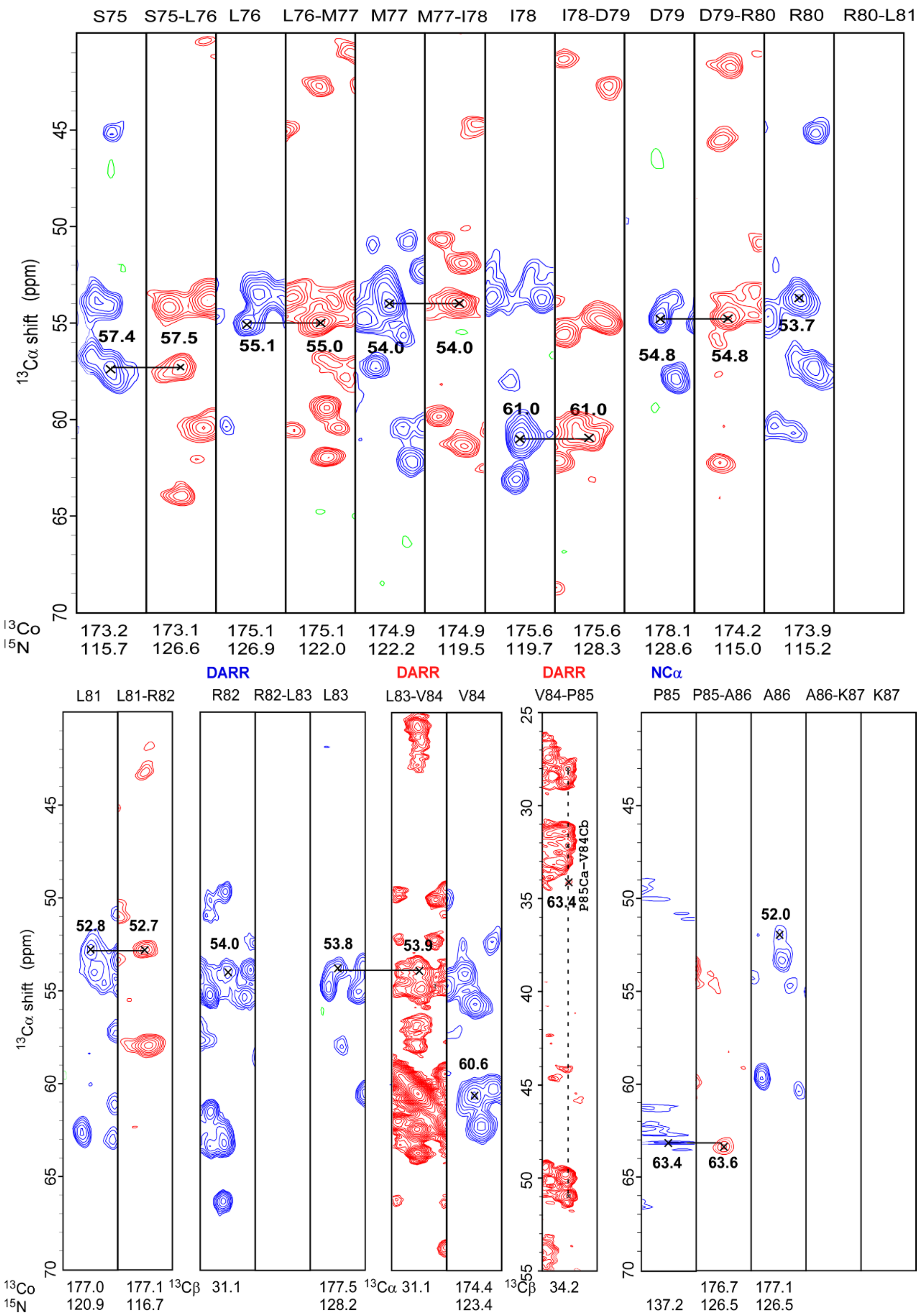

S75
S75-L76
L76
L76-M77
M77
M77-I78
I78
I78-D79
D79
D79-R80
R80
R80-L81
57.4
57.5
55.1
55.0
54.0
54.0
61.0
61.0
54.8
54.8
53.7
^{13}Cα shift (ppm)
^{13}Co
^{15}N
173.2 115.7
173.1 126.6
175.1 126.9
175.1 122.0
174.9 122.2
174.9 119.5
175.6 119.7
175.6 128.3
178.1 128.6
174.2 115.0
173.9 115.2
DARR
DARR
DARR
NCα
L81
L81-R82
R82
R82-L83
L83
L83-V84
V84
V84-P85
P85
P85-A86
A86
A86-K87
K87
52.8
52.7
54.0
53.8
53.9
60.6
63.4
P85Ca-V84Cb
52.0
63.4
63.6
177.0 120.9
177.1 116.7
^{13}Cβ 31.1
177.5 128.2
^{13}Cα 31.1
174.4 123.4
^{13}Cβ 34.2
137.2
176.7 126.5
177.1 126.5

Figure S5. Chemical shift perturbations plotted independently for X ≡ N, Cα, CO, Cβ. ΔδX=δ(bound gVp)-δ(free gVp).

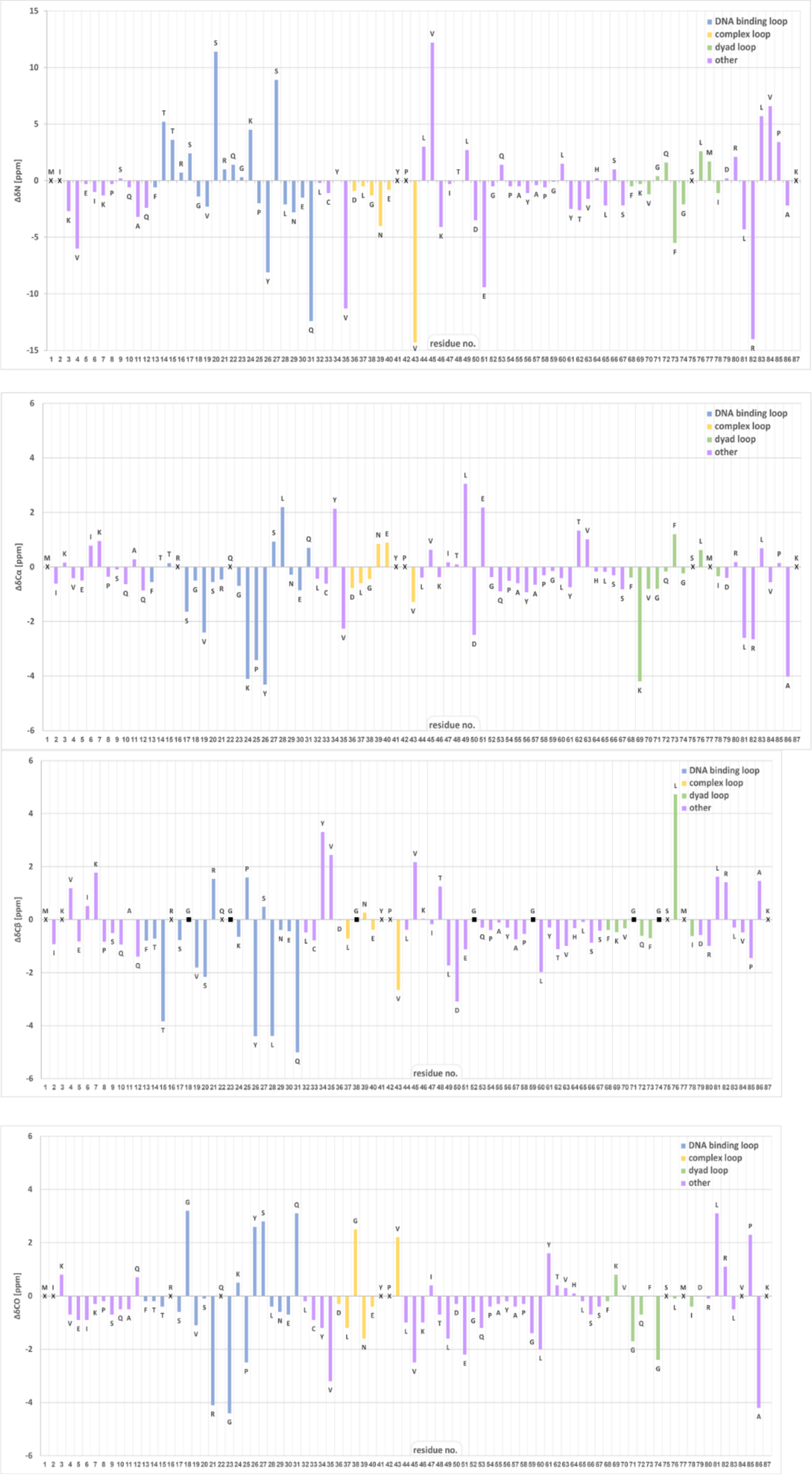

*Note that V43 and V45 are ambiguous in their assignment, and exchange of the two will cancel almost completely the CSPs of these two residues. In the main manuscript, we assumed they should be exchanged when calculating RMSD values.

Table S1. Chemical shift assignment of gVp bound to ssDNA. Deposited to BMRB accession number 51391. 'x' refers to unassigned atoms. '-' refers to non existing atoms.

	Cα	Cβ	Cδ/Cδ1	Cδ2	Cε/Cε1	Cε2	Cγ/Cγ1	Cγ2	CO	Cζ	N	Nδ1/Nδ2	Nε/Nε2	Nη1/Nη2	Nζ
**Met1**	55.8	x	-	-	x	-	x	-	177.6	-	x	-	-	-	-
**Ile2**	61.1	39.1	12.7	-	-	-	27.3	18.2	174.9	-	123.7	-	-	-	-
**Lys3**	56.8	33.2	x	-	41.7	-	25.2	-	175.1	-	127.9	-	-	-	x
**Val4**	60.2	35.8	-	-	-	-	23.2	21.7	173.3	-	123.3	-	-	-	-
**Glu5**	54.0	33.6	182.8	-	-	-	36.4	-	174.3	-	127.7	-	-	-	-
**Ile6**	59.6	38.4	12.6	-	-	-	28.2	16.5	178.0	-	126.2	-	-	-	-
**Lys7**	55.0	33.1	29.2	-	41.7	-	25.2	-	176.1	-	128.2	-	-	-	x
**Pro8**	66.4	31.4	49.7	-	-	-	28.2	-	178.5	-	135.0	-	-	-	-
**Ser9**	59.5	62.1	-	-	-	-	-	-	175.3	-	109.6	-	-	-	-
**Gln10**	55.0	28.8	x	-	-	-	35.8	-	176.0	-	122.1	-	109.1	-	-
**Ala11**	53.4	19.2	-	-	-	-	-	-	175.2	-	121.4	-	-	-	-
**Gln12**	53.9	30.0	180.2	-	-	-	32.8	-	174.7	-	114.1	-	112.4	-	-
**Phe13**	54.7	41.3	132.0	131.9	130.8	130.3	136.6	-	175.2	129.0	116.1	-	-	-	-
**Thr14**	60.8	70.9	-	-	-	-	-	21.8	173.9	-	114.4	-	-	-	-
**Thr15**	62.3	68.9	-	-	-	-	-	22.1	174.0	-	122.9	-	-	-	-
**Arg16**	53.9	33.0	x	-	-	-	27.1	-	173.5	157.1	127.7	-	x	x	-
**Ser17**	55.9	66.1	-	-	-	-	-	-	173.0	-	118.3	-	-	-	-
**Gly18**	45.3	-	-	-	-	-	-	-	175.1	-	106.7	-	-	-	-
**Val19**	58.0	34.2	-	-	-	-	21.8	19.5	173.1	-	116.4	-	-	-	-
**Ser20**	57.2	63.9	-	-	-	-	-	-	173.3	-	127.2	-	-	-	-
**Arg21**	53.8	30.6	43.1	-	-	-	27.8	-	177.5	159.2	126.4	-	x	x	-
**Gln22**	54.4	28.7	172.7	-	-	-	33.1	-	178.6	-	126.2	-	108.7	-	-
**Gly23**	45.1	-	-	-	-	-	-	-	171.3	-	108.8	-	-	-	-
**Lys24**	54.1	35.5	29.2	-	41.9	-	21.0	-	173.0	-	121.6	-	-	-	x
**Pro25**	60.6	34.4	49.4	-	-	-	27.1	-	175.0	-	137.6	-	-	-	-
**Tyr26**	54.0	41.2	131.8	134	x	x	x	-	180.1	x	120.7	-	-	-	-
**Ser27**	57.2	66.5	-	-	-	-	-	-	176.0	-	124.0	-	-	-	-
**Leu28**	55.7	41.4	26.1	22.14	-	-	27.2	-	174.2	-	123.3	-	-	-	-
**Asn29**	50.8	38.9	-	-	-	-	176.6	-	174.1	-	121.4	112.7	-	-	-
**Glu30**	53.8	33.5	183.7	-	-	-	35.9	-	174.6	-	120.2	-	-	-	-
**Gln31**	55.5	28.7	180.2	-	-	-	34.2	-	176.0	-	114.9	-	111.8	-	-
**Leu32**	53.9	43.4	25.6	22.84	-	-	27.2	-	173.7	-	127.3	-	-	-	-
**Cys33**	55.2	32.1	-	-	-	-	-	-	171.9	-	108.6	-	-	-	-
**Tyr34**	59.9	44.8	132.8	x	117.4	117.5	x	-	175.7	157.6	115.9	-	-	-	-
**Val35**	60.9	34.1	-	-	-	-	21.0	20.9	174.3	-	111.4	-	-	-	-
**Asp36**	53.4	40.8	-	-	-	-	180.5	-	174.6	-	126.2	-	-	-	-
**Leu37**	53.3	40.6	25.2	23.08	-	-	26.4	-	176.8	-	125.7	-	-	-	-
**Gly38**	45.2	-	-	-	-	-	-	-	175.8	-	106.7	-	-	-	-
**Asn39**	53.7	39.8	-	-	-	-	x	-	173.8	-	110.5	x	-	-	-
**Glu40**	61.0	29.0	183.4	-	-	-	35.9	-	175.6	-	119.7	-	-	-	-
**Tyr41**	55.7	41.5	133.1	x	117.5	x	x	-	177.5	157.6	123.1	-	-	-	-
**Pro42**	62.3	33.0	49.5	-	-	-	27.3	-	173.7	-	135.6	-	-	-	-
**Val43**	60.3	34.5	-	-	-	-	24.1	21.8	175.1	-	114.3	-	-	-	-
**Leu44**	55.0	41.7	26.5	23.46	-	-	27.9	-	174.5	-	128.3	-	-	-	-
**Val45**	60.7	36.8	-	-	-	-	23.9	23.3	172.8	-	128.4	-	-	-	-
**Lys46**	54.9	34.3	23.3	-	x	-	x	-	176.0	-	123.1	-	-	-	x
**Ile47**	60.5	40.7	13.9	-	-	-	27.5	18.4	176.1	-	128.1	-	-	-	-
**Thr48**	62.3	70.7	-	-	-	-	-	22.0	173.8	-	123.0	-	-	-	-
**Leu49**	57.9	41.7	x	24.18	-	-	26.9	-	177.3	-	129.9	-	-	-	-
**Asp50**	51.9	39.8	-	-	-	-	x	-	176.8	-	116.9	-	-	-	-
**Glu51**	60.5	28.6	182.8	-	-	-	36.5	-	175.4	-	113.5	-	-	-	-
**Gly52**	45.2	-	-	-	-	-	-	-	173.2	-	115.0	-	-	-	-
**Gln53**	52.4	29.5	179.7	-	-	-	32.2	-	173.3	-	122.1	-	110.0	-	-
**Pro54**	61.5	32.1	50.1	-	-	-	26.5	-	175.1	-	136.3	-	-	-	-
**Ala55**	52.2	18.1	-	-	-	-	-	-	179.0	-	119.9	-	-	-	-
**Tyr56**	59.5	38.0	132.6	131.5	x	x	130.6	-	176.7	156.4	123.0	-	-	-	-
**Ala57**	50.0	17.4	-	-	-	-	-	-	176.1	-	124.1	-	-	-	-
**Pro58**	63.3	31.3	49.8	-	-	-	28.7	-	175.9	-	132.6	-	-	-	-
**Gly59**	44.4	-	-	-	-	-	-	-	170.5	-	110.7	-	-	-	-
**Leu60**	53.7	42.7	24.8	23.03	-	-	27.1	-	176.8	-	119.1	-	-	-	-
**Tyr61**	57.2	45.1	132.2	132.7	117.4	117.5	x	-	175.4	157.6	121.9	-	-	-	-
**Thr62**	59.9	71.1	-	-	-	-	-	22.0	174.0	-	111.4	-	-	-	-
**Val63**	63.1	33.1	-	-	-	-	23.4	21.5	175.8	-	120.0	-	-	-	-
**His64**	57.9	32.7	-	118.8	138.8	-	136.6	-	177.6	-	129.7	x	x	-	-
**Leu65**	58.0	43.2	24.0	22.94	-	-	26.3	-	177.4	-	129.4	-	-	-	-
**Ser66**	60.5	62.5	-	-	-	-	-	-	176.0	-	116.6	-	-	-	-
**Ser67**	60.6	63.7	-	-	-	-	-	-	172.5	-	118.2	-	-	-	-
**Phe68**	56.8	41.4	132.0	132.2	131.6	x	139.7	-	174.1	128.5	116.4	-	-	-	-
**Lys69**	54.1	36.1	29.2	-	42.0	-	24.2	-	173.2	-	116.7	-	-	-	x
**Val70**	62.5	31.5	-	-	-	-	22.7	21.2	177.5	-	121.4	-	-	-	-
**Gly71**	44.8	-	-	-	-	-	-	-	175.6	-	114.2	-	-	-	-
**Gln72**	58.0	28.3	179.6	-	-	-	31.5	-	175.9	-	118.7	-	109.9	-	-
**Phe73**	59.7	37.9	132.4	134.6	x	x	x	-	176.4	x	111.8	-	-	-	-

**Gly74**	45.4	-	-	-	-	-	-	-	174.0	-	105.6	-	-	-	-
**Ser75**	57.4	64.1	-	-	-	-	-	-	173.2	-	115.4	-	-	-	-
**Leu76**	55.1	42.8	26.0	24.13	-	-	27.2	-	174.9	-	126.5	-	-	-	-
**Met77**	55.3	36.8	-	-	17.2	-	30.9	-	174.7	-	122.2	-	-	-	-
**Ile78**	61.1	38.0	12.5	-	-	-	29.1	16.5	175.7	-	119.5	-	-	-	-
**Asp79**	54.9	41.8	-	-	-	-	178.2	-	174.3	-	128.5	-	-	-	-
**Arg80**	54.0	33.2	x	-	-	-	x	-	174.0	x	115.1	-	x	x	-
**Leu81**	52.8	43.4	24.1	23	-	-	26.2	-	177.2	-	121.1	-	-	-	-
**Arg82**	54.1	31.1	x	-	-	-	25.2	-	176.7	166.6	116.4	-	x	x	-
**Leu83**	53.8	42.3	24.6	x	-	-	28.5	-	177.3	-	128.1	-	-	-	-
**Val84**	60.5	34.2	-	-	-	-	23.0	21.7	174.2	-	123.3	-	-	-	-
**Pro85**	63.4	32.1	51.0	-	-	-	28.0	-	176.8	-	137.2	-	-	-	-
**Ala86**	52.1	19.1	-	-	-	-	-	-	176.8	-	126.5	-	-	-	-
**Lys87**	x	x	x	-	x	-	x	-	x	-	x	-	-	-	x

Table S2: Experimental parameters of typical experiments

2D experiments:

Pulse sequence	DARR	DARR	DARR	DARR	DARR
Probe	Efree	Efree	Efree	Efree	Efree
Carbon isotopic labelling	U-[^{13}C,^{15}N]	U-[^{13}C,^{15}N]	[1,3-^{13}C]-Gly	[1,3-^{13}C]-Gly	[1,3-^{13}C]-Gly
^{1}H Frequency [MHz]	599.8	599.8	599.8	599.8	599.8
MAS rate (ω_r) [kHz]	13.000	13.000	13.000	13.000	13.000
Set temperature [℃]	-10.0	-10.0	-10.0	-10.0	-10.0
Carrier frequency [ppm]	100.15	100.15	99.99	99.99	99.99
Mixing time [ms]	15	100	50	100	300
Acquisition points(t_1/t_2)	1024/5988	1024/5988	1024/5988	1024/5988	1400/5988
Acquisition time (t_1/t_2) [ms]	13.11/29.94	13.13/29.94	13.13/29.94	13.13/29.94	17.95/29.94
Acquisition mode	States	States	States	States	States
$H90[\mu s]$	2.75	2.75	2.63	2.63	2.63
$C90[\mu s]$	5.00	5.00	5.00	5.00	5.00
CP power (v_H/v_C) [kHz]	79/50	79/50	68/50	68/50	68/50
CP contact time [ms] (10% linear ramp on ^{13}C)	1.8	1.8	1.2	1.2	1.2
^{1}H decoupling [kHz]	80	80	80	80	80
Relaxation delay [s]	3.6	3.6	3.2	3.6	3.2
Scans	16	16	16	16	16
SW (f_1/f_2) [kHz]	39/100	39/100	39/100	39/100	39/100

Pulse sequence	$CORD_{XY4}$	$CORD_{XY4}$
Probe	Efree	Efree
Carbon isotopic labelling	[1,3-^{13}C]-Gly	[1,3-^{13}C]-Gly
^{1}H Frequency [MHz]	599.8	599.8
MAS rate (ω_r) [kHz]	13	13
Set temperature [℃]	-10	-10
Carrier frequency [ppm]	99.87	99.87
Mixing time [ms]	150	300
Acquisition points(t_1/t_2)	1450/5988	1024/5988
Acquisition time (t_1/t_2) [ms]	18.58/29.94	13.13/29.94
Acquisition mode	States	States
$H90[\mu s]$	2.6	2.6
$C90[\mu s]$	5.0	5.0
CP power (v_H/v_C) [kHz]	63/50	63/50
CP contact time [ms] (10% linear ramp on ^{13}C)	1.2	1.2
^{1}H decoupling [kHz]	75	75
Relaxation delay [s]	3.5	3.2
Scans	32	32
SW (f_1/f_2) [kHz]	39/100	39/100

Pulse sequence (TBD)	NCO	NCA
Probe	Efree	Efree
Carbon isotopic labelling	U-[^{13}C,^{15}N]-Gly	U-[^{13}C,^{15}N]-Gly
^{1}H Frequency [MHz]	599.8	599.8

MAS rate (ω_r) [kHz]	13	13
Set temperature [℃]	-10	-10
Carrier frequency(N/C) [ppm]	122.7/175.0	122.6/60.1
Acquisition points(t_1/t_2)	160/5988	160/5988
Acquisition time (t_1/t_2) [ms]	12.3/29.94	12.3/29.94
Acquisition mode	States	States
$H90[\mu s]$	2.75	2.75
CP power (v_H/v_N) [kHz]	73/50	73/50
CP_{HN} contact time [ms] (10% linear ramp on ^{15}N)	1.25	1.25
DCP_{NC} contact time [ms]	3.5	2.5
^1H decoupling [kHz]	90	90
DCP condition (tangent ramp on ^{13}C)	$\omega_{15N}=1.5\omega_R$, $\omega_{13C}=2.5\omega_R$	$\omega_{15N}=2.5\omega_R$, $\omega_{13C}=1.5\omega_R$
Relaxation delay [s]	3.5	3.5
Scans	16	16
SW (f_1/f_2) [kHz]	6.5/100	6.5/100

Pulse sequence (TBD)	$NCOCX$	$NCACX$
Probe	Efree	Efree
Carbon isotopic labelling	U-[^{13}C,^{15}N]-Gly	U-[^{13}C,^{15}N]-Gly
^1H Frequency [MHz]	599.8	599.8
MAS rate (ω_r) [kHz]	13	13
Set temperature [℃]	-10	-10
Carrier frequency(N/C/C) [ppm]	122.7/175.0/100.2	122.7/60.1/100.2
DARR Mixing time [ms]	25	25
Acquisition points($t_1/t_2/t_3$)	90/64/4990	84/120/4990
Acquisition time ($t_1/t_2/t_3$) [ms]	10.4/9.8/24.95	9.7/9.2/24.95
Acquisition mode	States	States
$H90[\mu s]$	2.75	2.75
CP power (v_H/v_N) [kHz]	73/50	73/50
CP_{HN} contact time [ms] (10% linear ramp on ^{15}N)	1.25	1.25
DCP_{NC} contact time [ms]	3.5	2.5
^1H decoupling [kHz]	90	90
DCP condition (tangent ramp on ^{13}C)	$\omega_{15N}=1.5\omega_R$, $\omega_{13C}=2.5\omega_R$	$\omega_{15N}=2.5\omega_R$, $\omega_{13C}=1.5\omega_R$
Relaxation delay [s]	3	3
Scans	16	16
SW ($f_1/f_2/f_3$) [kHz]	4.33/3.25/100	4.33/6.5/100
